# A stepwise approach for the reproducible optimization of PAMO expression in *Escherichia coli* for whole-cell biocatalysis

**DOI:** 10.1186/1472-6750-12-31

**Published:** 2012-06-21

**Authors:** Edwin van Bloois, Hanna M Dudek, Wouter A Duetz, Marco W Fraaije

**Affiliations:** 1 Laboratory of Biochemistry, Groningen Biomolecular Sciences and Biotechnology Institute, University of Groningen, Nijenborgh 4, 9747 AG, Groningen, The Netherlands; 2 Enzyscreen BV, Tingietersweg 127, 2031 ER, Haarlem, The Netherlands

**Keywords:** Baeyer-Villiger monoxygenase, *Escherichia coli*, Biocatalysis, Square deep-well microtiter plates, Screening

## Abstract

**Background:**

Baeyer-Villiger monooxygenases (BVMOs) represent a group of enzymes of considerable biotechnological relevance as illustrated by their growing use as biocatalyst in a variety of synthetic applications. However, due to their increased use the reproducible expression of BVMOs and other biotechnologically relevant enzymes has become a pressing matter while knowledge about the factors governing their reproducible expression is scattered.

**Results:**

Here, we have used phenylacetone monooxygenase (PAMO) from *Thermobifida fusca*, a prototype Type I BVMO, as a model enzyme to develop a stepwise strategy to optimize the biotransformation performance of recombinant *E. coli* expressing PAMO in 96-well microtiter plates in a reproducible fashion. Using this system, the best expression conditions of PAMO were investigated first, including different host strains, temperature as well as time and induction period for PAMO expression. This optimized system was used next to improve biotransformation conditions, the PAMO-catalyzed conversion of phenylacetone, by evaluating the best electron donor, substrate concentration, and the temperature and length of biotransformation. Combining all optimized parameters resulted in a more than four-fold enhancement of the biocatalytic performance and, importantly, this was highly reproducible as indicated by the relative standard deviation of 1% for non-washed cells and 3% for washed cells. Furthermore, the optimized procedure was successfully adapted for activity-based mutant screening.

**Conclusions:**

Our optimized procedure, which provides a comprehensive overview of the key factors influencing the reproducible expression and performance of a biocatalyst, is expected to form a rational basis for the optimization of miniaturized biotransformations and for the design of novel activity-based screening procedures suitable for BVMOs and other NAD(P)H-dependent enzymes as well.

## Background

Over the last decade, enzymes have attracted much attention as they are efficient and extremely specific catalysts in many synthetic chemical applications. Baeyer-Villiger-monooxygenases (BVMOs) represent a notable example of a group of enzymes that have emerged as powerful biocatalysts [1]. BVMOs incorporate one atom of molecular oxygen into a carbon-carbon bond of an organic substrate next to a carbonyl group while the other oxygen atom is reduced to water. Most characterized BVMOs are NADPH-dependent flavoproteins and belong to a sequence-related family, called Type I BVMOs [2-4]. Phenylacetone monooxygenase (PAMO) from *Thermobifida fusca* represents a prototype Type I BVMO, and its characterization by us showed that it is a soluble, monomeric protein of about 65 kDa and is well expressed in *Escherichia coli*[5]. Substrate profiling revealed that it is mainly active towards small aromatic ketones and sulfides [5-7]. However, PAMO is also able to convert larger substrates, albeit with a poor activity and selectivity [8]. In addition, PAMO is remarkably thermostable and tolerant towards organic solvents [5,9,10]. The determination of its atomic structure showed that PAMO comprises two domains; an FAD and NADPH-binding domain with the active site sandwiched in between at the domain interface [11]. Moreover, a recent study, using complementary biochemical and structural experiments, revealed that PAMO and related enzymes function mainly as oxygen-activating enzymes. These can react with any appropriate substrate that is able to reach the catalytic center within the active site [12]. The detailed structural and mechanistic understanding of PAMO as well as its remarkable stability make this enzyme an attractive target for potential biocatalytic applications.

The reproducible expression of BVMOs and other biotechnologically relevant enzymes has become a pressing matter. Not only because of their growing use in a variety applications, but also in the design of novel screening methods for directed-evolution experiments to identify and isolate novel enzyme variants with the desired properties. Common strategies to optimize this typically rely on small scale reactions, using either purified enzyme, or whole cells expressing the enzyme of interest. Various studies on cyclohexanone monooxygenase (CHMO), a well-characterized BVMO from *Acinetobacter* sp.*,* demonstrate that whole cell biocatalytic systems are particularly well-suited for this purpose. Different whole cell biocatalytic systems, using *Saccharomyces cerevisiae* or *E. coli*, have been employed successfully to investigate and improve critical parameters for its expression as well as conditions for CHMO-catalyzed biotransformations [13-17]. Specifically, these systems were used either in microscale or bench-scale reactions for substrate profiling, analysis of substrate or product inhibition, comparison of different expression hosts, assessment of biocatalyst stability, analysis of oxygen supply, investigation of substrate uptake, quantification of kinetic data, and the detailed analysis of different microwell formats [15,17-24]. Combined, these studies emphasize the importance of a robust host organism in combination with a powerful expression system, and highlight the relevance of different factors governing the expression of the target enzyme, such as expression temperature, time and period of induction. Furthermore, they provide insight into conditions that control the efficiency of biotransformation, including the source of reducing power for *in vivo* coenzyme regeneration as well as substrate and product inhibition.

Although valuable, the overall picture provided by these studies is blurred because of the variety of host organisms, different expression systems, various model substrates and differing reaction conditions employed in several studies for the same biocatalyst. To provide a clear picture on this issue, we present in this study a rational and systematic approach to optimize the expression of a biocatalyst in a reproducible fashion. To this end, we have used PAMO as a model BVMO and followed a stepwise strategy to improve the biotransformation performance of recombinant *E. coli* expressing PAMO. Using a microscale approach, the best expression conditions for PAMO were investigated first, including different host strains, temperature as well as time and induction period for PAMO expression. Next, this optimized system was used to improve conditions of the biotransformation step, the PAMO-catalyzed conversion of phenylacetone, by evaluating the best electron donor, substrate concentration, and the temperature and length of biotransformation. This resulted in an efficient and highly reproducible PAMO whole cell biocatalyst and, moreover, the optimized procedure was successfully adapted for mutant screening. The strategy presented in this study provides a valuable tool for the reproducible optimization of bioconversions and in the design of novel activity-based screening procedures suitable for BVMOs and probably other NAD(P)H-dependent enzymes as well.

## Results and discussion

### Experimental approach

The optimization strategy presented in this study revolves around a recombinant *E. coli* strain expressing PAMO because a whole cell biocatalyst is an excellent system for this purpose as it is experimentally simple and the use of whole cells instead of the purified enzyme eliminates its costly isolation. To enable whole cell biocatalysis, we used an arabinose-inducible PAMO expression plasmid because the P_BAD_ promoter allows a tightly controlled and titratable overexpression unlike expression plasmids with a lac-type promotor [25].

Phenylacetone is the preferred substrate of PAMO and is converted into benzyl acetate [5]. This substrate was used as a model ketone throughout this study because we previously established that it is readily taken up by *E. coli* cells expressing PAMO and is converted into benzyl acetate with high efficiency [26]. Moreover, the formation of benzyl acetate by these cells can be quantitatively assayed by gas chromatography [5,26,27]. This procedure was, therefore, used to assess the effects of the different optimization steps on the activity of the PAMO whole cell biocatalyst. Furthermore, Stewart and co-workers have shown that non-growing cells are able to perform a CHMO-mediated model Beayer-Villiger oxidation more efficiently than growing cells [21]. Accordingly, we used non-growing cells for the PAMO-catalyzed biotransformation of phenylacetone.

In addition, all optimization steps were performed at a microscale level by using 96 square deep-well microtiter plates (96-sdMTPs) because this format is excellent for evaluating different conditions in parallel as well as bacterial growth [28]. All conditions experimentally addressed were evaluated on the basis of the rate with which benzyl acetate was formed during biotransformation and conditions yielding the best production were included for the next step.

### Best expression host, inducer concentration and expression temperature

**Figure 1:**
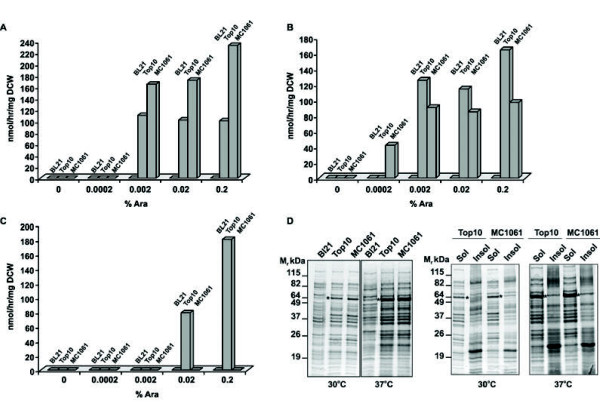
**Top10 is the best expression host in combination with 0.2% L-arabinose and 30°C for the production of PAMO.** The expression of PAMO was investigated in the standard expression strains Top10, MC1061, and BL21(DE)3 transformed with an arabinose-inducible PAMO expression plasmid. Cells were grown in the presence of increasing amounts of inducer (L-arabinose) in 96-sdMTP over night at 25°C (**A**), 30°C (**B**), or 37°C (**C**), respectively. The next day, cells were harvested and used for the bioconversion of phenylacetone in 96-sdMTP for 3 hours at 37°C followed by the analysis of the benzyl acetate content. All experiments were performed in duplicate and the values obtained were within 5% of each other. (**D**) PAMO expression in all tested strains. Cells were grown as described in the legend to Figure 1A at 30°C, or 37°C, respectively. Subsequently, a cell lysate was prepared, which was further separated into a soluble (sol) and insoluble fraction (insol) for Top10 and MC1061 by ultracentrifugation. Samples were analyzed by SDS-PAGE and Coomassie staining. The position of PAMO is indicated by an asterisk.

As a first step in our optimization strategy, we determined and improved critical factors that control the expression of PAMO. Out of these factors a powerful expression host is of key importance for high-level overexpression. *E. coli* is the most frequently used expression host primarily because of capability to produce recombinant proteins in high yields. However, it has been established that the production of the same target protein in various *E. coli* expression strains can differ dramatically [25,29]. Therefore, we established the best PAMO expression host out of three standard *E. coli* expression strains (Top10, MC1061 and BL21(DE3). Furthermore, the expression rate of the target protein is also determined by the inducer concentration (L-arabinose) and temperature [25,29], which were considered in our initial analysis as well. To study these parameters, cells of the aforementioned expression strains, harboring a PAMO expression plasmid, were grown to saturation in 96-sdMTP at 25, 30 or 37°C in the presence of increasing amounts of L-arabinose to induce PAMO expression. For subsequent biotransformations, cells were centrifuged and resuspended in assay mixture, containing 5 mM phenylacetone, and samples were incubated for 3 hours at 37°C. Following biotransformation, cells were removed by centrifugation, the supernatant was extracted with ethyl acetate and the amount of benzyl acetate was analyzed by GC. As shown in Figure 1, no production of benzyl acetate was detected when cells were grown in the absence of arabinose, indicating that background expression of PAMO is virtually absent in all strains. Similarly, no production of benzyl acetate was observed under all experimental conditions with BL21(DE3) as an expression host. In contrast, a significant formation of benzyl acetate was observed with Top10 and MC1061 grown at 25°C or 30°C (Figure 1 A and B) in the presence of 0.002-0.2% L-arabinose. At a growth temperature of 37°C, however, production of benzyl acetate was only observed for Top10 induced for PAMO expression with 0.02 or 0.2% L-arabinose (Figure 1 C).

To analyze the lack of product formation with BL21(DE3) and the contrasting results obtained with Top10 and MC1061 when grown at 37°C, we investigated the expression levels of PAMO in these strains. Therefore, cells were grown to saturation in 96-sdMTP at 30°C or 37°C in the presence of 0.2% arabinose to induce PAMO expression, a cell lysate of these cells was prepared and analyzed by SDS-PAGE (Figure 1 D left panel). This clearly showed that PAMO (indicated by an asterisk) was not expressed in BL21(DE3), thereby explaining the absence of benzyl acetate after biotransformation. This is probably caused by a poor induction of PAMO expression at 0.2% arabinose as BL21(DE3) is able to metabolize arabinose, which, may, therefore, impair induction of PAMO production. In contrast, PAMO was nicely expressed in Top10 and MC1061 when grown at both temperatures, which offered no explanation for the striking difference in the production of benzyl acetate. Although PAMO is expressed in MC1061 at 37°C, it is conceivable that PAMO is produced in a non-active form due to aggregation as insoluble inclusion bodies. Alternatively, the uptake of phenylacetone by MC1061 cells may be impaired after growth at this temperature. To distinguish between these two possibilities, the cell lyates prepared from Top10 and MC1061 cells were subjected to an ultracentrifugation step to obtain a soluble and insoluble fraction. SDS-PAGE analysis of these fractions showed that PAMO (indicated by an asterisk) was almost exclusively present in the soluble fraction of Top10 and MC1061 grown at 30°C or 37°C (Figure 1 D right panel). This, therefore, may suggest that benzyl acetate was not produced during biotransformation due to an impaired uptake of phenylacetone by MC1061 cells following growth at 37°C.

Based on these results, we decided to use Top10 as an expression host for PAMO considering its overall robust performance in combination with 0.2% L-arabinose and 30°C as standard conditions for expression in 96-sdMTP.

### Optimal induction time, induction period and effect of external riboflavin addition

**Figure 2:**
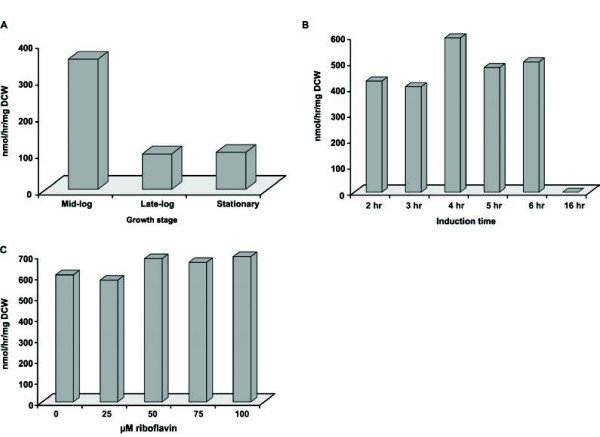
**The best activity of cells expressing PAMO is obtained with mid log cells, induced for PAMO expression for 4 hours and without external addition of riboflavin.** (**A**) Analysis of the best cellular growth stage for the induction of PAMO expression. Top10 cells were grown to mid log, late log or stationary phase as described in the Methods section, harvested and grown in the presence of 0.2% L-arabinose in 96-sdMTP for 3 hours at 30°C. Following PAMO expression, cells were used for the bioconversion of phenylacetone in 96-sdMTP as described in the legend to Figure 1. (**B**) Assessment of the best induction time for the production of PAMO. Top 10 cells were grown to mid log phase and used for the production of PAMO in 96-sdMTP as described in the legend to Figure 2A. Starting 2 hours after induction, samples were removed hourly and used for the bioconversion of phenylacetone in 96-sdMTP for 2 hours at 37°C followed by the analysis of the benzyl acetate content. (**C**) Effect of external riboflavin addition. Mid log Top10 cells were induced for PAMO expression as described in the legend to Figure 2A in the presence of the indicated amounts of riboflavin. Following PAMO expression, cells were used for the bioconversion of phenylacetone as described in the legend to Figure 1 followed by the analysis of the benzyl acetate content. All experiments were performed in duplicate and the values obtained were within 5% of each other.

It has been established that there is a tight correlation between the production of recombinant proteins by *E. coli* and the time of induction e.g. the cellular growth stage at which induction is initiated. For example, it appears advantageous to induce the expression of the target protein when cells have entered the log phase because at this stage cells are rapidly growing, which requires a highly active translation machinery and this can be exploited for the high-level production of recombinant proteins [25,29]. However, several studies show that the latter can also be obtained with late log or stationary phase cells, displaying a (greatly) reduced growth rate [30-32]. We, therefore, investigated the optimal induction time for PAMO expression. To this end, Top10 cells harboring a PAMO expression plasmid were grown to OD_660_ values of 0.4, 0.8 or 3.0, corresponding to mid log phase, late log phase or stationary phase, respectively. Aliquots of these cells were removed and induced for PAMO expression with 0.2% L-arabinose at 30°C in 96-sdMTP for 3 hours. Following PAMO expression, cells were harvested and used for the biotransformation of phenylacetone as described above. Analysis of benzyl acetate production revealed that late log and stationary phase cells displayed a poor production of benzyl acetate unlike mid log cells (Figure 2A). This is consistent with the results from other studies, showing that the log phase is the preferred time point to start the production of recombinant proteins in *E. coli*[25,29].

Next, we studied the length of the induction period because this is of significance with respect to high-level overexpression of target proteins. To analyze the best induction period for the expression of PAMO, Top10 cells were grown to mid log phase and induced for PAMO expression at 30°C in 96-sdMTP. Cells were collected, starting 2 hours after induction, at 1 hour intervals and used for the bioconversion of phenylacetone after which the benzyl acetate content was analyzed (Figure 2B). This showed that the production of benzyl actetate was relatively constant up to 6 hours after induction. However, 4 hours of induction resulted in its best formation, whereas benzyl acetate was no longer formed after 16 hours of induction probably due to a loss of PAMO expression.

It has been reported that exogenously added riboflavin, an FAD precursor which is taken up by *E. coli* unlike FAD, improves the activity of different heterologously expressed flavoproteins like pyridoxine 4-oxidase from *Microbacterium luteolum*[33]. Therefore, we studied whether the performance of our PAMO whole cell biocatalyst could be improved by the addition of riboflavin during the induction phase. As shown in Figure 2C, the production of benzyl acetate was not significantly improved by increasing amounts of riboflavin, suggesting that sufficient FAD is available within *E. coli* cells to sustain a proper PAMO expression.

Collectively, these data show that PAMO expression should be initiated by addition of L-arabinose at mid log phase for a period of 4 hours. Moreover, the addition of riboflavin is not required to improve the activity of our whole cell system.

### Best biotransformation conditions

**Figure 3:**
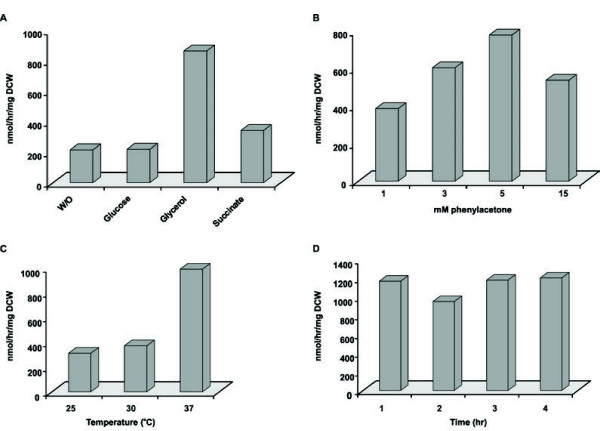
**The highest biocatalytic performance is achieved with glycerol as electron donor in combination with 5 mM phenylacetone and 37°C for biotransformation.** (**A**) Effect of externally added carbohydrates as source of reducing power. Phenylacetone was converted by Top10 cells expressing PAMO in the presence of glucose, glycerol, or succinate after which the benzyl acetate content was analyzed. (**B**) Analysis of the optimal substrate concentration. Top10 cells producing PAMO were used for the conversion of the indicated amounts of phenylacetone followed by analysis of benzyl acetate production. (**C**) Influence of biotransformation temperature. Phenylacetone was converted by Top10 cells expressing PAMO at 25°C, 30°C, or 37°C followed by analysis of the benzyl acetate content. (**D**) Assessment of best biotransformation period. Top10 cells producing PAMO were used for the bioconversion of phenylacetone and the formation of benzyl acetate was analyzed at 1 hour intervals. For all panels, cells were grown as described in the legend to Figure 2A, and biotransformations were performed as described in the legend to Figure 1. All experiments were performed in duplicate and the values obtained were within 5% of each other.

We next analyzed and improved basic conditions for the biotransformation step, using our recombinant *E. coli* strain in combination with the optimized expression protocol from the previous stage. BVMOs are typically NADPH-dependent and require an efficient system for cofactor regeneration. To this end, several elegant solutions have been presented that circumvent the addition of costly cofactors and work well for cell-free systems and purified enzymes. These include a new generation of self-sufficient BVMO systems, comprising a fusion between a thermostable variant of phosphite dehydrogenase and different BVMOs [34,35]. However, many BVMO-based whole cell systems rely on *in vivo* coenzyme regeneration by the host, which can be improved by coexpression of glucose-6-phosphate dehydrogenase or external addition of carbohydrates [18-22,24]. We preferred to explore the latter approach as it is experimentally simpler than coexpression of glucose-6-phosphate dehydrogenase or photochemical coenzyme regeneration [36,37]. Therefore, we investigated the effect of different externally added carbohydrates (glucose, glycerol and succinate) on the biocatalytic performance of our PAMO whole cell system. These carbohydrates were added during biotransformation, after which their effect on the production of benzyl acetate was evaluated (Figure 3A). This revealed that addition of glucose or succinate hardly improved the biocatalytic performance when compared to the negative control that did not contain any externally added source of reducing power. Remarkably, the addition of glycerol quadrupled the production of benzyl acetate by our whole cell system relative to the negative control, indicative of efficient coenzyme regeneration upon addition of glycerol as shown before [15,18,19]. Moreover, our data indicate that glucose and succinate are not efficiently utilized by *E. coli* for the regeneration of NADPH unlike glycerol. Possibly, these carbohydrates serve other metabolic purposes in addition to biotransformation-related NADPH regeneration. The latter is consistent with a recent study involving a recombinant *E. coli* strain expressing the *Pseudomonas* sp. styrene monooxygenase genes *styAB* and glucose as a source of reducing power. These were employed to show that biocatalysis-related NAD(P)H consumption of this system was unexpected high, thereby pointing towards other metabolic roles of glucose during redox biocatalysis [38].

Furthermore, we investigated the effect of increasing amounts of phenylacetone on the activity of our PAMO whole cell system because it was recently shown that high concentrations of relevant substrates were deleterious for the biocatalytic activity of other BVMO whole cell systems [18,22,24]. To analyze this, cells expressing PAMO were resuspended in an assay mix containing increasing concentrations of phenylacetone and following biotransformations, the benzyl acetate content of these samples was assessed (Figure 3B). This showed that 15 mM of phenylacetone impairs the production of benzyl acetate. In contrast, the performance of our whole cell system increased substantially when 3 or 5 mM of phenylacetone were used as evidenced by the higher production of benzyl acetate under these conditions. In addition, we also analyzed whether the production of benzyl acetate could be improved by increasing the amount of cells for biotransformation. This revealed that the best formation of benzyl acetate was obtained with 0.1 mg DCW and, moreover, its production was adversely affected by increasing the amount of cells (data not shown).

It has been well established that the temperature is a highly significant factor influencing the activity of enzymes and therefore also of whole cell biocatalytic systems [39]. Consequently, we investigated the effect of the temperature on the biotransformation activity of our whole cell system by performing the bioconversion of phenylacetone at 25, 30, and 37°C. As shown in Figure 3C, the production of benzyl acetate was relatively moderate at 25 and 30°C. At 37°C, however, a three-fold increase in the formation of benzyl acetate was obtained, which is reflective of the optimal temperature of *E. coli* and increased phenylacetone monooxygenase activity.

Finally, we sought to identify the best biotransformation period in order to obtain the best production of benzyl acetate. For this purpose, we performed a time course experiment in which the production of benzyl acetate by our whole cell biocatalyst was analyzed at 1 hour intervals (Figure 3D). This revealed that the amount of benzyl acetate increased almost linearly over time for up to 4 hours, indicating that its formation rate was remarkably constant during this period.

Combined, these data suggest that glycerol is the best external source of reducing power for the regeneration of NADPH during the PAMO-catalyzed biotransformation of phenylacetone. Furthermore, the best biocatalytic performance was observed at 37°C in combination with 5 mM of substrate. In contrast, the performance of our PAMO whole cell biocatalyst was strongly affected by decreasing the temperature, or increasing the substrate concentration as well as the amount of cells for biotransformation.

### Efficiency of PAMO whole cell biocatalyst

After having established the best conditions for expression and biotransformation, we next wished to assess the efficiency of our PAMO whole cell biocatalyst. To this end, Top10 cells expressing PAMO were grown under optimized conditions in 96- sdMTP and after 4 hours of induction cell samples were collected. Subsequently, samples were analyzed by SDS-PAGE and Coomassie staining after which the amount of PAMO was quantified by gel band volume analysis. This revealed that 730 ng (12 pmol) of PAMO was produced by 1 OD_660_ unit of *E. coli* Top10 cells. Theoretically, 12 pmol PAMO is able to produce 130 nmol of benzyl acetate per hour given its *k*_cat_ of 3 s^-1^ for phenylacetone [27]. This theoretical production rate compares favorably with the experimentally determined formation rate of 117 nmol of benzyl acetate per hour and shows that the biocatalytic performance of our whole cell system is most likely not impaired by oxygen transfer and substrate accessibility as suggested for other whole-cell systems [23,40]. As noted above, the biocatalytic performance was adversely affected by increasing the amount of cells for biotransformation which may point towards a limited oxygen transfer under these conditions.

### Analysis of reproducibility and use in activity-based mutant screening

**Figure 4:**
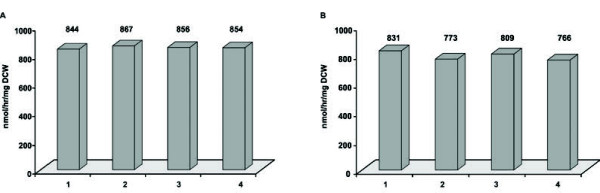
**The biocatalytic performance of cells expressing PAMO is highly reproducible.** All optimized parameters for expression and biotransformation were combined and phenylacetone was converted by Top10 cells producing PAMO washed with buffer (**B**) or not washed (**A**) prior to biotransformation. Cells were grown as described in the legend to Figure 2A, and biotransformations were performed as described in the legend to Figure 1.

Reproducibility is an important requirement for an (industrial) biocatalytic process and in the design of novel screening procedures for directed-evolution experiments. We, therefore, studied first the reproducibility of the biocatalytic performance of our whole cell system. To this end, all optimized parameters for the expression as well as biotransformation were combined and biotransformations were performed in fourfold using either washed *E. coli* cells expressing PAMO (Figure 4B) or non-washed cells (Figure 4A). Subsequent analysis of the benzyl acetate content revealed that the production of this compound by cells that were washed with buffer prior to biotransformation was slightly less when compared to cells that did not receive this treatment as evidenced by a benzyl acetate productivity of 795 nmol/hr/mg DCW for non-washed cells and 855 nmol/hr/mg DCW for washed cells. Moreover, the results obtained were highly reproducible as indicated by the relative standard deviation of 1% for non-washed cells and 3% for washed cells.

Encouraged by these results, we wished to determine next if our optimized protocol could be adapted successfully for screening purposes. Therefore, we investigated whether *E. coli* cells expressing the previously described PAMO mutant M446G [27] could be distinguished from cells expressing the wild-type enzyme based on their activity towards 1-indanone when all optimized conditions for expression and biotransformation were combined compared to the same set-up under non-optimized conditions (e.g. stationary phase cells in combination with 0.02% L-arabinose and 37°C for protein expression in 96-sdMTP). Of note, it has been established that 1-indanone is not converted by wild-type PAMO unlike the M446G variant, resulting in the unexpected lactone 1-isochromanone [26,41]. Cells expressing the PAMO mutants P440L and P440N were included as an additional control for specificity of the procedure because of their broadened substrate scope and therefore potential activity with 1-indanone [42]. Following biotransformation, the 1-isochromanone content in all samples was analyzed by GC, showing that 1-indanone was not converted by the wild-type enzyme like the P440L and P440N variant under all conditions tested. However, 1-isochromanone was detected following bioconversion of 1-indanone by cells expressing the M446G mutant as expected. Interestingly, a two-fold increase in the amount of 1-isochromanone was obtained when cells producing the M446G variant were subjected to the optimized protocol relative to non-optimized conditions. Importantly, the observed lack of 1-indanone conversion is not caused by a poor expression because all PAMO variants were equally well expressed under these experimental conditions (data not shown), thereby pointing towards a low reactivity of PAMO P440L and P440G with 1-indanone like the wild-type enzyme and possibly a lowered uptake of the substrate under non-optimized conditions. These data, nevertheless, emphasize the potential of our optimized procedure in the design of novel activity-based screening procedures for BVMOs and other NAD(P)H-dependent enzymes.

## Conclusions

Driven by the growing demand for the reproducible expression of biocatalysts, we provide here a comprehensive overview of key factors that control the reproducibility and performance of a whole cell biocatalyst. Using recombinant *E. coli* producing PAMO, we have developed a stepwise strategy to optimize the expression of PAMO in a reproducible fashion. This system was first used to explore the parameters that are of importance for PAMO expression like: host strain, inducer concentration, temperature as well as time and length of induction. Furthermore, this whole cell system was used to improve biotransformation conditions by evaluating the best electron donor, substrate concentration, and the temperature and length of biotransformation. Our results show that the type of expression host, cellular growth stage at which induction is initiated and the length of the induction period are amongst the most important factors that control the expression of PAMO. In addition, we found that the type of carbohydrate used as a source of reducing power (for regeneration of NAD(P)H) during biotransformation and temperature are crucial for a high biocatalytic performance. Specifically, a more than four-fold enhancement of the biocatalytic performance was obtained when all optimized parameters were combined, which was highly reproducible as indicated by the relative standard deviation of 1% for non-washed cells and 3% for washed cells. Of note, additional factors known to influence the biocatalytic performance of a whole cell system such as, for example, medium composition, coexpression of chaperones, oxygen transfer, and substrate accessibility [23,40,43] were not considered in this study and therefore further improvement of our whole cell system is conceivable. Moreover, we show here that our optimized procedure can be adapted for activity-based screening procedures for BVMOs.

In summary, the optimization strategy presented here provides a clear picture on crucial factors controlling the reproducible expression and performance of a whole cell biocatalyst. Therefore, it is expected to form a rational basis for the optimization of biotransformations and for the design of novel activity-based screening procedures suitable for BVMOs and probably other NAD(P)H-dependent enzymes as well.

## Methods

### Enzymes, chemicals and media

Restriction enzymes were from Roche applied science and New England Biolabs. Pfu Turbo DNA polymerase was purchased from Stratagene. All chemicals were obtained from ACROS organics, Jülich Fine Chemicals, Roche Applied Sciences, and Sigma-Aldrich. Bacto tryptone and yeast extract, which were used for the preparation of media, were purchased from Becton, Dickinson & Company. All strains were routinely grown in Luria Bertani medium (LB; per liter, 10 g tryptone, 5 g yeast extract, 5 g NaCl) under aerobic conditions unless indicated otherwise. Where appropriate, ampicillin (100 μg/ml) was added to the culture medium.

### Strains and plasmids

Strain TOP10 (Invitrogen) was used as a routine host for all plasmid constructs. Strains Top10, MC1061 [44], and BL21(DE3) [45] were used for whole cell biotransformations in 96-sdMTP. The arabinose-inducible expression plasmid pPAMO [5] was used for the expression of PAMO in all strains. The previously described PAMO mutants M446G, P440N, and P440L were used for screening purposes [27,42]. All PAMO mutants were obtained by site-directed mutagenesis, using the QuikChange kit (Stratagene) and pPAMO as template. Nucleotide sequences were verified by DNA sequencing (GATC, Konstanz). Primer sequences are available upon request.

### Biomass conversions

Biomass concentrations were analyzed spectrophotometrically (Hitachi U-1100) at 660 nm and converted to dry cell weight (DCW) using the equation 1 OD_660_ = 0.3 g DCW/L [46,47].

### *Whole cell biotransformations in 96-*sdMTP

For whole cell biotransformations, cells were typically grown to saturation at 37°C and back-diluted 1:100 into fresh LB containing appropriate antibiotics. These cultures were grown to an OD_660_ value of 0.4 (corresponding to mid log phase) at 17 °C overnight. The following day, 1 OD_660_ unit of cells was harvested and resuspended in 800 μl of fresh LB, containing appropriate antibiotics and 0.2% L-arabinose to induce the expression of PAMO. Cultures were grown for 4 hours in 96-sdMTP (Waters, 2 ml square collection plate) at 30°C in a Titramax 1000 shaker (Heidolph) at 1050 rpm, 1.5 mm shaking diameter. Subsequently, cells were harvested and resuspended in 500 μl phosphate-buffered saline, containing 10 mM glycerol, 5 mM phenylacetone, or 5 mM 1-indanone for screening purposes. Bioconversions were performed in 96-sdMTP (Waters, 2 ml square collection plate) for 3 hours at 37°C with shaking essentially as described [26]. Following bioconversions, cells were removed by centrifugation and samples were processed and analyzed by gas chromatography as described [26,27]. Unless indicated otherwise, all experiments were performed in duplicate and the values obtained were within 5% of each other.

### Cell fractionations and SDS-PAGE

Cells were grown to saturation at 37°C overnight and the next day back-diluted 1:100 into fresh LB containing appropriate antibiotics and 0.2% L-arabinose to induce the expression of PAMO. Cultures were grown in 96-sdMTP as described above and following the expression of PAMO, cells were harvested and resuspended in 1 ml of 50 mM Tris–HCl, pH = 7.5. This cell suspension was subjected to two brief rounds of sonication, followed by a clarifying spin to obtain a clarified cell lysate. This lysate was further fractionated into a soluble and insoluble fraction by ultracentrifugation (100,000 x *g* for 30 min at 4°C). Cellular fractions were normalized on the basis of the OD_660_ and samples of these fractions, containing equal OD_660_ units, were analyzed on standard 12% SDS-PAGE gels followed by Coomassie blue staining to visualize protein bands.

## Abbreviations

BVMO, Baeyer-Villiger monooxygenases; CHMO, Cyclohexanone monooxygenases; PAMO, Phenylacetone monooxygenases; SdMTP, Square deep-well microtiter plates.

## Competing interests

Dr. Wouter A. Duetz has a financial interest in Enzyscreen BV.

## Authors’ contributions

EB performed the experiments and analyzed the experimental data, and drafted the manuscript. HD, WAD and MWF have made substantial contributions to conception, interpretation of data and revised the manuscript. All authors read and approved the final manuscript.
